# Influence of Depression on the Quality of Life in Patients With Parkinson's Disease in Southwest Nigeria

**DOI:** 10.7759/cureus.65077

**Published:** 2024-07-22

**Authors:** Obinna James Orji, Chimaobi Ezekiel Ijioma, Jeffrey Ehiedu Odarah, Izuchukwu Elvis Okeji, Joseph Ekama Areh, Donatus Onyebuchi Anele, Innocent Chima Zacs, Emmanuel Ayomide Kalesanwo, Ochuko Austin-Jemifor, Uchenna Chika Nnamani

**Affiliations:** 1 Department of Acute Medicine, University Hospital of Derby and Burton, NHS Foundation Trust, Derby, GBR; 2 Department of Pediatrics, Abia State Specialist Hospital and Diagnostics Centre, Umuahia, NGA; 3 Department of Geriatrics, JBS Gerontology Centre and Medicare Services, Lekki, NGA; 4 Department of General Medicine, North Cumbria Integrated Care, NHS Foundation Trust, North Cumbria, GBR; 5 Department of Emergency Medicine, Warrington and Halton Hospitals, NHS Foundation Trust, Warrington, GBR; 6 Department of Pharmacology and Therapeutics, College of Medicine and Health Sciences, Gregory University, Uturu, NGA; 7 Department of Neurology, Ladoke Akintola University of Technology (LAUTECH) Teaching Hospital, Ogbomoso, NGA; 8 Department of Internal Medicine, Abia State University Teaching Hospital, Aba, NGA; 9 Department of Surgery, Olabisi Onabanjo University Teaching Hospital, Sagamu, NGA; 10 Department of Internal Medicine, Queen Elizabeth Hospital Birmingham, University Hospitals Birmingham, NHS Foundation Trust, Birmingham, GBR; 11 Department of General Practice, General Hospital, Umunze, NGA

**Keywords:** southwest, neurodegenerative disorder, quality of life, depression, parkinson's disease

## Abstract

Background

Parkinson's disease (PD) is a chronic neurodegenerative disorder that significantly impacts patients' quality of life (QoL). Depression is a common comorbidity in patients with PD, potentially exacerbating QoL deterioration. This study aimed to assess the influence of depression on QoL in patients with PD at Ladoke Akintola University of Technology (LAUTECH), Ogbomoso, Nigeria.

Methodology

A cross-sectional, descriptive study design was utilized. The study included 420 patients with PD attending the Neurology Clinic at LAUTECH. A purposive sampling technique was employed to select participants. Data collection instruments included the Unified Parkinson's Disease Rating Scale (UPDRS) for PD assessment, the Hoehn and Yahr scale for PD staging, the Patient Health Questionnaire-9 (PHQ-9) for depression evaluation, and the Parkinson's Disease Questionnaire-39 (PDQ-39) for QoL assessment. Data were analyzed using SPSS version 25.0 (IBM Corp., Armonk, NY), with descriptive and inferential statistics (Chi-square test) to determine associations, considering a *P*-value < 0.05 as significant.

Results

Among the participants, the prevalence of moderate to severe depression was 245, representing 58.81%. QoL assessment revealed that 297 (70.71%) patients with PD reported poor to very poor overall QoL. A significant association was found between the degree of depression and overall QoL (*P* = 0.000). Patients with severe depression reported the poorest QoL, while those with minimal to no depression reported the highest QoL scores.

Conclusions

Depression significantly impacts the QoL in patients with PD at LAUTECH in southwest Nigeria. Addressing depression in PD management is crucial to improve patient outcomes.

## Introduction

Parkinson's disease (PD) is a progressive neurodegenerative disorder characterized by motor symptoms such as bradykinesia, rigidity, resting tremor, and postural instability, as well as a variety of nonmotor symptoms including depression, cognitive impairment, and sleep disturbances [1]. Depression is particularly common among patients with PD, with prevalence estimates ranging from 20% to 50% [2]. The presence of depression in PD can significantly worsen the quality of life (QoL) of patients, making it an important area of study, especially in diverse socioeconomic and cultural contexts like Nigeria.

QoL in patients with PD is a multifaceted construct that encompasses physical health, psychological state, level of independence, social relationships, personal beliefs, and relationships to salient features of the environment [3]. The motor symptoms of PD alone can drastically reduce QoL, leading to physical disability and social isolation. Nonmotor symptoms, particularly depression, further exacerbate these challenges by reducing patients' motivation to engage in social and physical activities, thus creating a detrimental cycle of worsening health and declining QoL [4].

Depression in PD is often underdiagnosed and undertreated due to overlapping symptoms between depression and PD, such as psychomotor slowing and fatigue [5]. Moreover, depression in patients with PD may present atypically, with symptoms like anxiety, irritability, and somatic complaints being more prominent than in the general population [6]. The etiology of depression in PD is multifactorial, involving neurobiological, psychological, and social factors. Neurobiologically, depression in PD is linked to dopaminergic, serotonergic, and noradrenergic dysfunctions [7].

Numerous studies have demonstrated that depression is a strong predictor of reduced QoL in patients with PD, often more so than the severity of motor symptoms [8]. Depression can impair cognitive function, increase disability, and lower overall life satisfaction. Additionally, depression in PD is associated with higher healthcare utilization and costs due to increased morbidity and the need for more intensive care [9].

Research on PD in African countries, including Nigeria, is limited. However, the available data suggest that patients with PD in Nigeria face unique challenges that may influence the prevalence and impact of depression on QoL. These include limited access to healthcare services, stigma associated with both PD and mental health disorders, and socioeconomic constraints [10]. In Nigeria, cultural perceptions and traditional beliefs about disease and mental health can also affect how symptoms are reported and treated. In a setting where an illness is still highly stigmatized symptoms of such illness might be embarrassing and so more likely to be denied [11].

Given the high prevalence of depression among patients with PD and its significant impact on QoL, it is important to understand this relationship in the Nigerian context to develop effective interventions. This study aims to fill the gap in the literature by examining the influence of depression on QoL in patients with PD attending Ladoke Akintola University of Technology (LAUTECH) Teaching Hospital in Ogbomoso, Nigeria. By doing so, it seeks to contribute to improved clinical management and policy-making that can enhance the well-being of patients with PD in Nigeria and similar settings.

## Materials and methods

Study design

This research adopted a cross-sectional, descriptive study design to assess the influence of depression on the QoL in patients with PD.

Study setting

The study was conducted at LAUTECH Teaching Hospital, Ogbomoso, Nigeria. This hospital serves as a tertiary healthcare facility and receives referrals for PD management.

Study population

The study population comprised patients diagnosed with PD attending the Neurology Clinic at LAUTECH Teaching Hospital.

Sample size determination

The sample size was determined using Fisher’s formula outlined by Airaodion et al. [12]:

n = ((Z^2 (Pq))/e^2)

where n = minimum sample size

Z = 1.96 at 95% confidence level,

P = known prevalence of depression in PD in Nigeria

e = error margin tolerated at 5% = 0.05

q = 1 - p

According to Ojo et al. [13], the existing prevalence of depression in patients with PD in Nigeria was 55%:

P = 55% = 0.55

q = 1 - p

= 1 - 0.55

= 0.45

n = ((1.96)^2 (0.55 x 0.45))/ (0.05)^2)

n = (3.8416 x 0.2475)/(0.0025)

n = 380.3184

The minimum sample size was 380 and was adjusted to 420 to account for a nonresponse rate of 10 %.

Sampling technique

A purposive sampling technique was used to select participants. All patients with PD who met the inclusion criteria during the study period (April 2023 to March 2024) were included in the study.

Inclusion criteria

It included patients diagnosed with PD based on clinical criteria, attending regular follow-up at LAUTECH, aged 40 years and above, and providing informed consent to participate in the study.

Exclusion criteria

It included patients with other neurodegenerative diseases as well as those who did not consent to participate.

Data collection instruments

These included:

Sociodemographic data: This section included questions on age, sex, educational level, and marital status.

Assessment of PD: The Unified Parkinson's Disease Rating Scale (UPDRS) was used to evaluate the severity of PD symptoms, both motor and nonmotor.

Evaluation of stages of PD: The Hoehn and Yahr Staging of PD was used to evaluate the stages of PD.

Assessment of depression: The Patient Health Questionnaire-9 (PHQ-9) was utilized to assess the prevalence and severity of depression.

Assessment of QoL: The Parkinson's Disease Questionnaire-39 (PDQ-39) was used to evaluate various dimensions of QoL in patients with PD.

Data collection procedure

Recruitment

Patients attending the Neurology Clinic were informed about the study. Those who consented were enrolled.

Interview and Questionnaire Administration

Trained research assistants conducted face-to-face interviews and administered the questionnaires. Each interview took approximately 45 minutes.

Data management and analysis

Data were entered into a secure database and analyzed using SPSS software version 25.0 (IBM Corp., Armonk, NY). Descriptive statistics (frequency and percentage) were summarized: sociodemographic characteristics, disease severity, depression levels, and QoL. Inferential statistics (Chi-square test) was used to evaluate the association between depression and QoL, with a *P*-value < 0.05 considered statistically significant.

Ethical considerations

Ethical approval was obtained from the Ethical Review Committee of LAUTECH Teaching Hospital, Ogbomoso, with approval number LTH/OGB/EC/2023/196. Informed consent was obtained from all participants after explaining the purpose and procedures of the study. Confidentiality and anonymity of participants were strictly maintained throughout the study.

## Results

This study involved 420 respondents. Most participants were aged 70 and above (255, 60.71%), followed by those aged 60-69 (105, 25.00%), 50-59 (49, 11.67%), and 40-49 (11, 2.62%), as presented in Table 1. The majority were male (238, 56.67%), with females making up 182 (43.33%). Educational levels varied, with 234 (55.71%) having secondary education, 127 (30.24%) having tertiary education, 42 (10.00%) having primary education, and 17 (4.05%) having no formal education. Most respondents were divorced or widowed (254, 60.48%), followed by married (164, 39.05%) and single (2, 0.48%) (Table 1).

**Table 1:** Sociodemographic details of respondents.

Variable	Frequency (*n* = 420)	Percentage (%)
Age (in years)		
40 – 49	11	2.62
50 – 59	49	11.67
60 – 69	105	25.00
70 and above	255	60.71
Sex		
Male	238	56.67
Female	182	43.33
Educational level		
No formal education	17	4.05
Primary education	42	10.00
Secondary education	234	55.71
Tertiary education	127	30.24
Marital status		
Single	02	0.48
Married	164	39.05
Divorced/Widowed	254	60.48

The results in Table 2 show that respondents reported varying degrees of difficulty with nonmotor and motor experiences of daily living. For activities like dressing and hygiene, 106 (25.24%) experienced severe difficulty. Mood fluctuations were severe for 123 (29.29%). Cognitive impairments like memory problems were moderate for 303 (72.14%) and severe for 76 (18.10%). Tremors at rest were moderate for 276 (65.71%) and severe for 88 (20.95%). Bradykinesia was severe in 144 (34.29%) of cases. Muscle rigidity was severe for 131 (31.19%). Motor examinations revealed severe difficulties in finger tapping (186, 44.29%), hand movements (148, 35.24%), and leg agility (149, 35.48%).

**Table 2:** Assessment of Parkinson's disease. Assessment of Parkinson's disease was done using the Unified Parkinson's Disease Rating Scale (UPDRS). Data are presented as *n* (%). Not at all = 0, Mild = 1, Moderate = 2, Severe = 3.

Variable	Not at all	Mild	Moderate	Severe
Nonmotor experiences of daily living				
Do you experience difficulty with activities of daily living, such as dressing, hygiene, eating, or using utensils?	18 (4.29)	72 (17.14)	224 (53.33)	106 (25.24)
Are you experiencing fluctuations in mood, including depression, anxiety, or apathy?	08 (1.90)	94 (22.38)	195 (46.43)	123 (29.29)
Do you have any cognitive impairments, such as memory problems, difficulty concentrating, or confusion?	04 (0.95)	37 (8.81)	303 (72.14)	76 (18.10)
Motor experiences of daily living				
Do you experience tremors at rest?	02 (0.47)	54 (12.86)	276 (65.71)	88 (20.95)
Rate the severity of bradykinesia (slowness of movement) in daily activities such as walking, dressing, or eating.	09 (2.14)	72 (17.14)	195 (46.43)	144 (34.29)
Do you experience muscle rigidity or stiffness that affects your mobility or range of motion?	07 (1.67)	99 (23.57)	183 (43.57)	131 (31.19)
Motor examination				
Finger tapping: Has tapping your index finger and thumb together for 30 seconds reduced?	03 (0.71)	104 (24.76)	121 (28.81)	186 (44.29)
Hand movements: Are there any difficulties in rapidly turning your hand from palm up to palm down?	07 (1.67)	111 (26.43)	154 (36.67)	148 (35.24)
Leg agility: Has tapping your foot for 30 seconds reduced?	06 (1.43)	114 (27.14)	151 (35.95)	149 (35.48)

**Figure 1:**
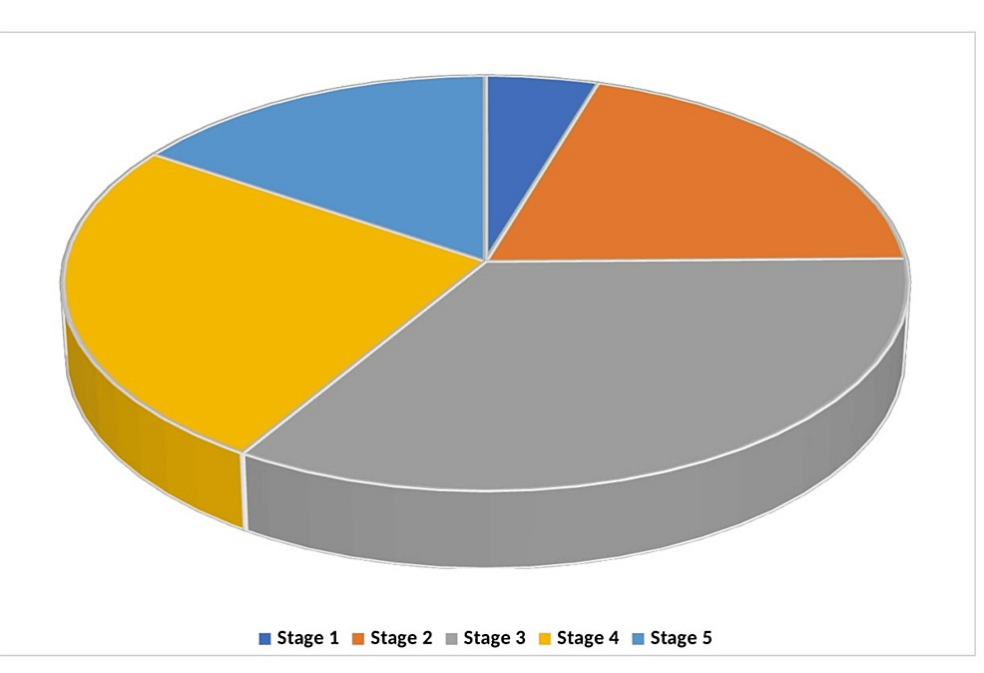
Stages of Parkinson’s disease of participants. Data are presented in %. Stages of Parkinson’s Disease was grouped using the Hoehn and Yahr Staging of Parkinson's disease. Stage 1 = Unilateral involvement only. Stage 2 = Bilateral or midline involvement without impairment of balance. Stage 3 = Bilateral involvement with impaired balance. Stage 4 = Severe disability, but still able to walk or stand unassisted. Stage 5 = Wheelchair-bound or bedridden unless aided.

The findings presented in Figure 1 reveal that most participants were in Stage 3 (143, 34.05%), followed by Stage 4 (106, 25.48%), Stage 2 (84, 20.00%), Stage 5 (66, 15.71%), and Stage 1 (20, 4.76%).

Little interest or pleasure in activities was experienced nearly every day by 93 (22.14%) of respondents, as presented in Table 3. Feelings of being down, depressed, or hopeless were experienced nearly every day by 78 (18.57%). Sleep issues were frequent, with 216 (51.43%) experiencing trouble for several days. Fatigue was nearly daily for 108 (25.71%), and poor appetite or overeating was a problem for 212 (50.48%) respondents on more than half the days. Concentration troubles were significant, with 112 (26.67%) experiencing them nearly every day (Table 3).

**Table 3:** Assessment of depression. Assessment of depression was done using the Patient Health Questionnaire-9 (PHQ-9). Data are presented as *n* (%). Not at all = 0, Several days = 1, More than half the days = 2, Nearly every day = 3.

Variable	Not at all	Several days	More than half of the days	Nearly every day
Little interest or pleasure in doing things	64 (15.24)	142 (33.81)	121 (28.81)	93 (22.14)
Feeling down, depressed, or hopeless	102 (24.29)	129 (30.71)	111 (26.43)	78 (18.57)
Trouble falling or staying asleep, or sleeping too much	44 (10.48)	216 (51.43)	106 (25.24)	54 (12.86)
Feeling tired or having little energy	28 (6.67)	118 (28.10)	166 (39.52)	108 (25.71)
Poor appetite or overeating	38 (9.05)	124 (29.52)	212 (50.48)	46 (10.95)
Feeling bad about yourself or that you are a failure or have let yourself or your family down	51 (12.14)	131 (31.19)	150 (35.71)	88 (20.95)
Trouble concentrating on things, such as reading the newspaper or watching television	13 (3.10)	167 (39.76)	128 (30.48)	112 (26.67)
Moving or speaking so slowly that other people could have noticed? Or the opposite - being so fidgety or restless that you have been moving around a lot more than usual	38 (9.05)	155 (36.90)	131 (31.19)	96 (22.86)
Thoughts that you would be better off dead or hurting yourself in some way	85 (20.24)	140 (33.33)	136 (32.38)	59 (14.05)

**Figure 2:**
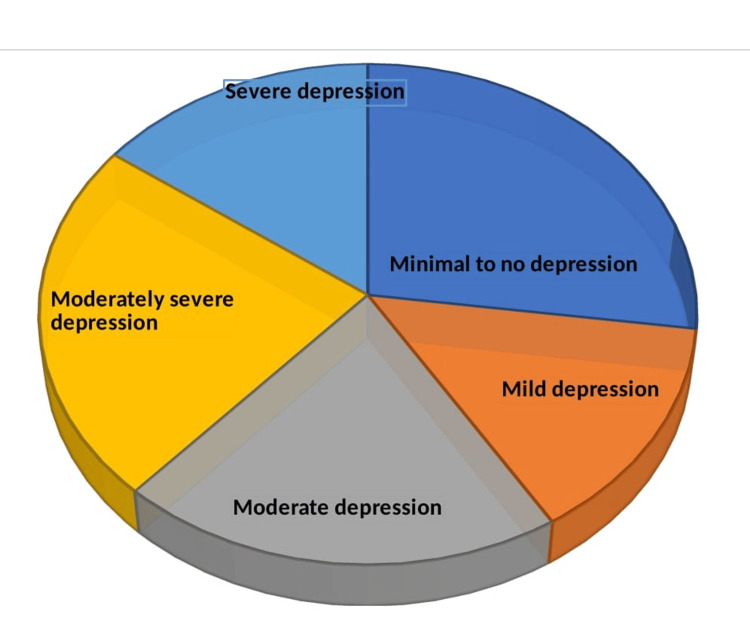
Degree of depression among patients with Parkinson’s disease. Data are presented in %. Degree of depression was estimated using the Patient Health Questionnaire-9 (PHQ-9) scores. 0-4 = Minimal to no depression. 5-9 = Mild depression. 10-14 = Moderate depression. 15-19 = Moderately severe depression. 20-27 = Severe depression.

The PHQ-9 scores indicated that 114 (27.14%) had minimal to no depression, 59 (14.05%) had mild depression, 86 (20.48%) had moderate depression, 98 (23.33%) had moderately severe depression, and 63 (15.00%) had severe depression, as shown in Figure 2.

Difficulty turning over in bed was experienced often by 89 (21.19%), as presented in Table 4. Getting out of bed was an issue for 93 (22.14%). Walking difficulties were frequent, with 134 (31.90%) experiencing them often. Freezing of gait occurred often for 138 (32.86%). Daily activities like cutting food and needing assistance were issues, with 127 (30.24%) and 121 (28.81%) reporting frequent difficulties, respectively. Emotional well-being concerns were common, with 111 (26.43%) feeling depression often. Stigma was also significant, with 123 (29.29%) often feeling embarrassed in social situations (Table 4).

**Table 4:** Assessment of quality of life of patients with Parkinson’s disease. The Parkinson's Disease Questionnaire (PDQ-39) was used for assessing QoL in patients with PD. Data are presented as *n* (%). Never = 0, Rarely = 1, Occasionally = 2, Often = 3, Always = 4.

Variable	Never	Occasionally	Sometimes	Often	Always
Mobility					
How often do you experience difficulty turning over in bed?	84 (20.00)	123 (29.29)	102 (24.29)	89 (21.19)	22 (5.24)
How often do you have trouble getting out of bed?	62 (14.76)	119 (28.33)	115 (27.38)	93 (22.14)	31 (7.38)
In the past month, how often have you experienced difficulty walking due to Parkinson's disease?	31 (7.38)	63 (15.00)	128 (30.48)	134 (31.90)	64 (15.24)
How often do you find yourself avoiding going out due to concerns about your ability to walk?	29 (6.90)	58 (13.81)	134 (31.90)	136 (32.38)	63 (15.00)
How often do you experience freezing of gait?	38 (9.05)	62 (14.76)	122 (29.05)	138 (32.86)	60 (14.29)
Activities of daily living					
How often do you have difficulty cutting your food?	41 (9.76)	79 (18.81)	118 (28.10)	127 (30.24)	55 (13.10)
How often do you need assistance with activities such as dressing, bathing, or eating due to Parkinson's disease?	36 (8.57)	82 (19.52)	110 (26.19)	121 (28.81)	71 (16.90)
How much does Parkinson's disease affect your ability to perform household chores?	29 (6.90)	62 (14.76)	133 (31.67)	108 (25.71)	88 (20.95)
How often do you feel frustrated or limited by your ability to perform daily tasks independently?	34 (8.10)	65 (15.48)	120 (28.57)	122 (29.05)	79 (18.81)
Emotional well-being					
How often do you experience feelings of depression or sadness related to Parkinson's disease?	71 (16.90)	63 (15.00)	97 (23.10)	111 (26.43)	78 (18.57)
How often do you feel anxious or worried about your condition?	72 (17.14)	60 (14.29)	100 (23.81)	109 (25.95)	79 (18.81)
Stigma					
How often do you feel embarrassed in social situations because of your Parkinson's disease symptoms?	22 (5.24)	83 (19.76)	91 (21.67)	123 (29.29)	101 (24.05)
How often do you feel others treat you differently because of your Parkinson's disease?	26 (6.19)	83 (19.76)	93 (22.14)	119 (28.33)	99 (23.57)
How often do you avoid social situations due to concerns about how others perceive your condition?	28 (6.67)	85 (20.24)	101 (24.05)	114 (27.14)	92 (21.90)
Social support					
How often do you feel supported by your family and friends in dealing with your Parkinson's disease?	08 (1.90)	31 (7.38)	61 (14.52)	147 (35.00)	173 (41.19)
How often do you feel isolated or lonely because of your Parkinson's disease?	173 (41.19)	147 (35.00)	61 (14.52)	30 (7.14)	09 (2.14)
How often do you participate in activities or groups with others who have Parkinson's disease for support?	18 (4.29)	37 (8.81)	82 (19.52)	156 (37.14)	127 (30.24)
Cognitive impairment					
How often do you have difficulty remembering things?	16 (3.81)	31 (7.38)	55 (13.10)	195 (46.43)	123 (29.29)
How often do you have trouble concentrating?	14 (3.33)	29 (6.90)	81 (19.29)	161 (38.33)	135 (32.14)
How often do cognitive issues impact your daily functioning or decision-making?	16 (3.81)	38 (9.05)	77 (18.33)	160 (38.10)	129 (30.71)
Communication					
How often do you have difficulty speaking clearly?	33 (7.86)	64 (15.24)	81 (19.29)	124 (29.52)	118 (28.10)
How often do you struggle to find the right words?	19 (4.52)	41 (9.76)	66 (15.71)	146 (34.76)	148 (35.24)
Have you experienced difficulties being understood by others?	08 (1.90)	22 (5.24)	49 (11.67)	208 (49.52)	133 (31.67)
Bodily discomfort					
How often do you experience pain because of your Parkinson's disease?	02 (0.48)	31 (7.38)	44 (10.48)	176 (41.90)	167 (39.76)
How often do you feel physically uncomfortable because of your Parkinson's disease?	02 (0.48)	31 (7.38)	44 (10.48)	176 (41.90)	167 (39.76)
To what extent does bodily discomfort interfere with your ability to enjoy daily activities?	02 (0.48)	31 (7.38)	44 (10.48)	176 (41.90)	167 (39.76)

**Figure 3:**
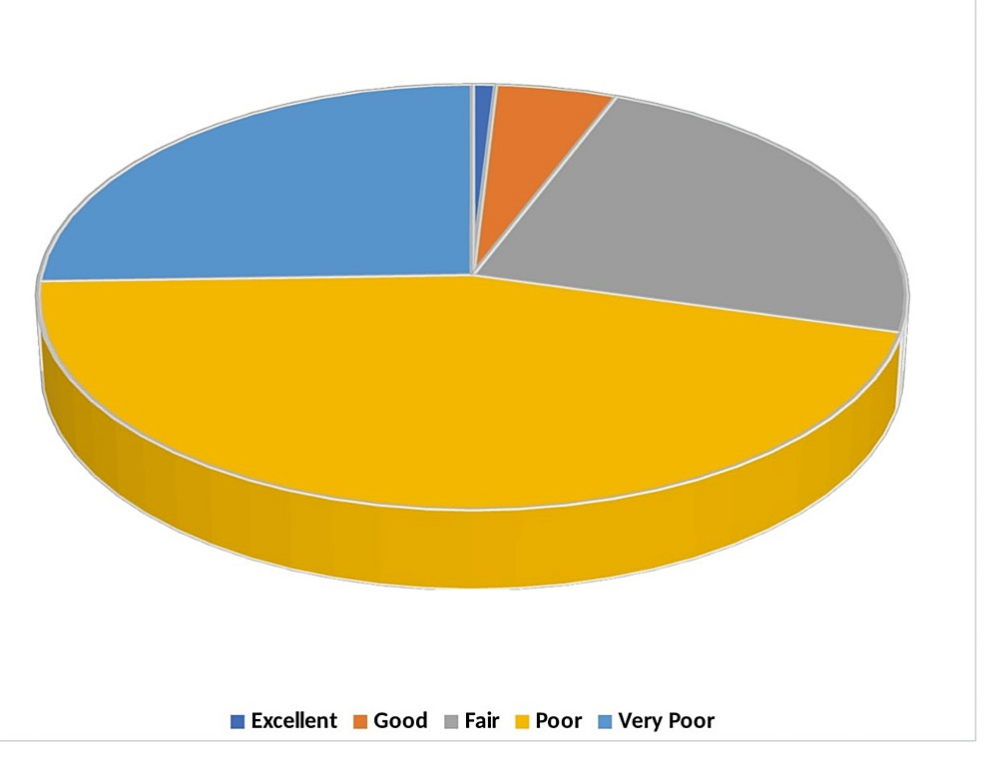
Overall quality of life of patients with Parkinson’s disease. Data are presented in %.

Figure 3 shows that the overall QoL was poor for 190 (45.24%) of respondents, very poor for 107 (25.48%), fair for 98 (23.33%), good for 21 (5.00%), and excellent for only 4 (representing 0.95% of the participants).

The outcome of the influence of depression on the QoL in Parkinson's disease patients presented in Table 5 revealed that depression significantly influenced the QoL (*P* < 0.0001). Those with minimal to no depression had a better QoL, with 19 (16.67%) rating it as good and 88 (77.19%) as fair. Severe depression had the most negative impact, with 63 (100%) of those affected rating their QoL as very poor.

**Table 5:** Influence of depression on the quality of life in patients with Parkinson's disease. Data are presented as *n* (%). *P*-value < 0.05 is considered statistically significant.

Degree of depression	Overall quality of life					*X*^2^	*P*-value
	Excellent	Good	Fair	Poor	Very Poor		
Minimal to no depression	04 (3.51)	19 (16.67)	88 (77.19)	03 (2.63)	00 (0.00)	9.183	0.000
Mild depression	00 (0.00)	02 (3.39)	09 (15.25)	48 (81.36)	00 (0.00)		
Moderate depression	00 (0.00)	00 (0.00)	01 (1.16)	83 (96.51)	02 (2.33)		
Moderately severe depression	00 (0.00)	00 (0.00)	00 (0.00)	56 (57.14)	42 (42.86)		
Severe depression	00 (0.00)	00 (0.00)	00 (0.00)	00 (0.00)	63 (100.00)		

## Discussion

PD is a progressive neurodegenerative disorder characterized by both motor and nonmotor symptoms. The impact of PD on patients' QoL is profound, and the presence of depression can exacerbate this impact. This study assessed the influence of depression on the QoL among patients with PD attending the LAUTECH Teaching Hospital, Ogbomoso, Nigeria.

The UPDRS assessment revealed significant variations in the experiences of patients with PD (Table 2). These findings align with previous research indicating that motor and nonmotor symptoms significantly impair the QoL in patients with PD. Studies by Hely et al. and Shulman et al. similarly reported high prevalence rates of motor symptoms, such as bradykinesia and rigidity, and nonmotor symptoms, such as depression and cognitive impairments [14,15].

Most of the participants in this study were found to be in stages 3-5 of PD (Figure 1). This reflects a significant portion of patients in advanced stages of the disease, which correlates with increased disability and reliance on assistance for daily activities. The prevalence of advanced stages (Stages 3-5) in this study mirrors findings from other populations. For instance, a study by Muangpaisan et al. reported similar stage distributions, with a significant proportion of patients in the mid to late stages of the disease [16]. This stage progression is critical as it impacts the management strategies and the psychological well-being of patients.

Depression is a common nonmotor symptom of PD that significantly affects the QoL. In this study, nearly three-quarters of patients reported moderate-to-severe mood fluctuations, including depression. The high prevalence of depression in patients with PD is consistent with other studies. For instance, a systematic review by Reijnders et al. found that up to half of patients with PD suffer from depressive symptoms [2].

Depression in patients with PD can exacerbate the burden of both motor and nonmotor symptoms, leading to a reduced QoL. Depression has been associated with increased disability, poorer cognitive function, and reduced overall life satisfaction [4]. This study's findings highlight the necessity of addressing depressive symptoms as part of a comprehensive PD management plan to improve patients' QoL.

The findings from this study corroborate previous research indicating a high burden of both motor and nonmotor symptoms among patients with PD, with depression playing a significant role in deteriorating their QoL. For example, a study by Aarsland et al. found that depression significantly impacts the QoL in patients with PD, even more so than the severity of motor symptoms [17]. Similarly, Ravina et al. highlighted that depressive symptoms in patients with PD were associated with greater disability and a higher burden of nonmotor symptoms [18].

Examining the degree of depression (Figure 2) underscores the significant mental health burden borne by patients with PD at the LAUTECH Teaching Hospital. The findings of this present study are consistent with previous literature that underscores the high prevalence of depression in patients with PD. The Parkinson's Outcomes Project, the largest clinical study of PD, found depression to be one of the most common nonmotor symptoms affecting patients, with significant impacts on the QoL [19].

Furthermore, a study by Schrag et al. highlighted that depression in patients with PD often goes under-recognized and undertreated, contributing to a substantial decrease in the QoL [4]. This observation is mirrored in the present study, where a notable proportion of patients experience moderate to severe depression, indicating a need for improved mental health screening and intervention strategies.

A substantial proportion reported poor or very poor QoL, indicating severe impairments. Only a small fraction rated their QoL as excellent or good. From the influence of depression on QoL, patients with minimal to no depression mostly reported fair QoL, and only a small fraction reported poor QoL. In contrast, as the severity of depression increased, the QoL drastically deteriorated. Patients with mild depression predominantly reported poor QoL, while those with moderate to severe depression reported a combination of poor and very poor QoL. Notably, all patients with severe depression reported very poor QoL, demonstrating a direct correlation between depression severity and decreased QoL (*P* < 0.0001).

The findings of this study align with existing literature that underscores the significant impact of depression on the QoL of patients with PD. For instance, Schrag et al. noted that depression is a major predictor of poor QoL in patients with PD, affecting emotional well-being, social interactions, and overall life satisfaction [4]. Similarly, a study by Aarsland et al. highlighted that depression and anxiety are among the most debilitating nonmotor symptoms in PD, profoundly impairing daily functioning and QoL [17].

Moreover, recent studies have continued to emphasize the relationship between depression and QoL in PD. Dissanayaka et al. demonstrated that depressive symptoms significantly correlate with lower QoL scores, particularly in domains related to emotional and social functioning [20]. Additionally, Broen et al. found that addressing depressive symptoms through appropriate interventions can lead to marked improvements in QoL among patients with PD [21].

Limitations of the study

The study utilized a cross-sectional design, which captured a snapshot of the relationship between depression and quality of life at a single point in time. This design did not allow for the determination of causality or the assessment of changes over time. Data on depression and quality of life were collected using self-reported questionnaires. This method was subject to biases such as social desirability bias, recall bias, and subjective interpretation of the questions, especially considering the large number of patients with apparent cognitive impairment. These biases could affect the accuracy of the responses and, consequently, the study’s findings. Conducting the study in a single tertiary health facility may have limited the diversity of the patient population. Patients attending this facility might have had different characteristics or access to resources compared to those attending other facilities, influencing the study’s findings.

## Conclusions

This study reveals a significant association between depression and the QoL among patients with PD at LAUTECH Teaching Hospital in Ogbomoso, Nigeria. As the severity of depression increases, there is a significant decline in the overall QoL among patients with PD. Specifically, patients with minimal to no depression exhibit a notably better QoL compared to those with mild, moderate, moderately severe, or severe depression. This underscores the important role of addressing mental health issues, such as depression, in improving the well-being and QoL of individuals managing PD. These findings emphasize the importance of integrating mental health support alongside medical interventions for comprehensive patient care and improved outcomes.
